# Diagnostic performance of magnetic resonance imaging features to differentiate adrenal pheochromocytoma from adrenal tumors with positive biochemical testing results

**DOI:** 10.1186/s12880-024-01350-0

**Published:** 2024-07-18

**Authors:** Rukun Huang, Tingsheng Lin, Mengxia Chen, Xiaogong Li, Hongqian Guo

**Affiliations:** 1 Department of Urology, Affiliated Drum Tower Hospital, Medical School of Nanjing University, Nanjing, China; 2 Institute of Urology, Nanjing University, Nanjing, China

**Keywords:** Pheochromocytoma, Adrenal tumor, Magnetic Resonance Imaging (MRI), Hormone, Catecholamine

## Abstract

**Background:**

It is extremely essential to accurately differentiate pheochromocytoma from Adrenal incidentalomas (AIs) before operation, especially biochemical tests were inconclusive. We aimed to evaluate the value of magnetic resonance imaging (MRI) features to differentiate pheochromocytomas among adrenal tumors, among which the consequences of biochemical screening tests of catecholamines and/or catecholamine metabolites are positive.

**Methods:**

With institutional review board approval, this study retrospectively compared 35 pheochromocytoma (PHEO) patients with 27 non-pheochromocytoma(non-PHEO) patients between January 2022 to September 2023, among which the consequences of biochemical screening tests of catecholamines and/or catecholamine metabolites are positive. T test was used for the independent continuous data and the chi-square test was used for categorical variables. Univariate and multivariate logistic regression were applied to find the independent variate of the features to differentiate PHEO from non-PHEO and ROC analysis was applied to evaluate the diagnostic value of the independent variate.

**Results:**

We found that the T2-weighted (T2W) signal intensity in patients with pheochromocytoma was higher than other adrenal tumors, with greatly significant (p < 0.001). T2W signal intensity ratio (T2W nodule-to-muscle SI ratio) was an independent risk factor for the differential diagnosis of adrenal PHEOs from non-PHEOs. This feature alone had 91.4% sensitivity and 81.5% specificity to rule out pheochromocytoma based on optimal threshold, with an area under the receiver operating characteristics curve (AUC‑ROC) of 0.910(95% C I: 0.833–0.987).

**Conclusion:**

Our study confirms that T2W signal intensity ratio can differentiate PHEO from non-PHEO, among which the consequences of biochemical screening tests of catecholamines and/or catecholamine metabolites are positive.

**Supplementary Information:**

The online version contains supplementary material available at 10.1186/s12880-024-01350-0.

## Introduction

Adrenal incidentalomas (AIs) were detected to indicate that there is a rising clinical trouble with the augmenting use of abdominal computed tomography (CT), and magnetic resonance imaging (MRI) [1]. Adrenal tumors, which account for roughly 5–7% of the adult people [2], are generally nonfunctioning-adrenocortical adenomas (AAs); however, it may also be diseases that required deeper examination and special clinical treatment (e.g., pheochromocytomas(PHEOs), adrenocortical carcinoma, or metastatic lesions) [3]. The main clinical issue to be determined in this setting is the hormonal activity of these lesions [4]. Catecholamines are usually produced in PHEOs, causing typical symptoms such as hypertension. Most pheochromocytoma can be diagnosed by clinical symptoms while 5–58% of cases can be asymptomatic [5, 6]. Therefore, it is extremely crucial to accurately differentiate pheochromocytoma from Adrenal incidentalomas (AIs) before operation, although pheochromocytoma (PHEO) represents less than 5% of all adrenal incidentalomas [7]. Accurate preoperative identification of pheochromocytoma is essential for appropriate treatment planning. Unrecognized pheochromocytomas are associated with high mortality, with the most common complication being cardiovascular disease [8, 9]. It is known to all that biochemical screening tests of catecholamines and/or catecholamine metabolites play essential roles (“golden standard”) in the diagnosis of PHEOs. Biochemical testing is advised for the workup of incidental adrenal nodules with reported accuracy of over 90% for diagnosis of pheochromocytoma [1, 3, 10]. However, the measurement of these hormones and metabolites is expensive, cumbersome, and time-consuming, and can be confounded by multiple medications and dietary components [11]. And, these tests may be falsely negative or falsely positive [12]. Therefore, imaging examinations, especially MRI, are extremely vital in clinical practice and have an irreplaceable function in the diagnosis of PHEOs, particularly in the cases that the biochemical screening tests come back false positive.

It is reported that the radiological features of the adrenal incidentalomas have been proved to be very precise in determining whether the adrenal lesion is a PHEO or not [13–16]. Washout CT were reported to have a greatly specific value for differentiating adenomas from non-adenomas [16–18]. However, they are limited by a heterogeneous control group of “non-adenomas” [19, 20], and there is overlap in the imaging features of pheochromocytoma and adenoma compared at CT washout [17, 21, 22]. PHEOs misdiagnosis as lipid-poor adenomas (LPAs) emerged based on washout criteria in a number of cases [17, 18]. In patients received MRI, the diagnosis of adrenal adenomas depends on the ability to record microscopic fats in lipid-rich adenomas by quantitative signal strength (SI) ratios measured using dual-echo chemical shift MRI [23, 24]. However, it has been reported that approximately 30% of adrenal adenomas are lipid-poor [25]. Previous investigators have shown that pheochromocytoma tend to be of higher T2-weighted SI compared with adenomas [26]. Nevertheless, the characteristic T2-weighted (T2W) hyperintensity is not present in approximately 30% of pheochromocytomas [27].

However, it is rarely reported that the diagnostic ability of multiparameter magnetic resonance in distinguishing pheochromocytoma from adenoma, especially the consequences of biochemical screening tests of catecholamines and/or catecholamine metabolites are positive. The purpose of the present study was therefore to evaluate the multiparameter MRI for the differentiation of PHEO from adrenal adenomas, in a population of pheochromocytomas and adenomas with positive biochemical results and to evaluate accuracy of diagnosis. This method is used to compensate for the inaccurate detection of catecholamines and metabolites, so as to improve the diagnostic accuracy of pheochromocytoma in clinical practice.

## Materials and methods

### Patients

**Fig. 1:**
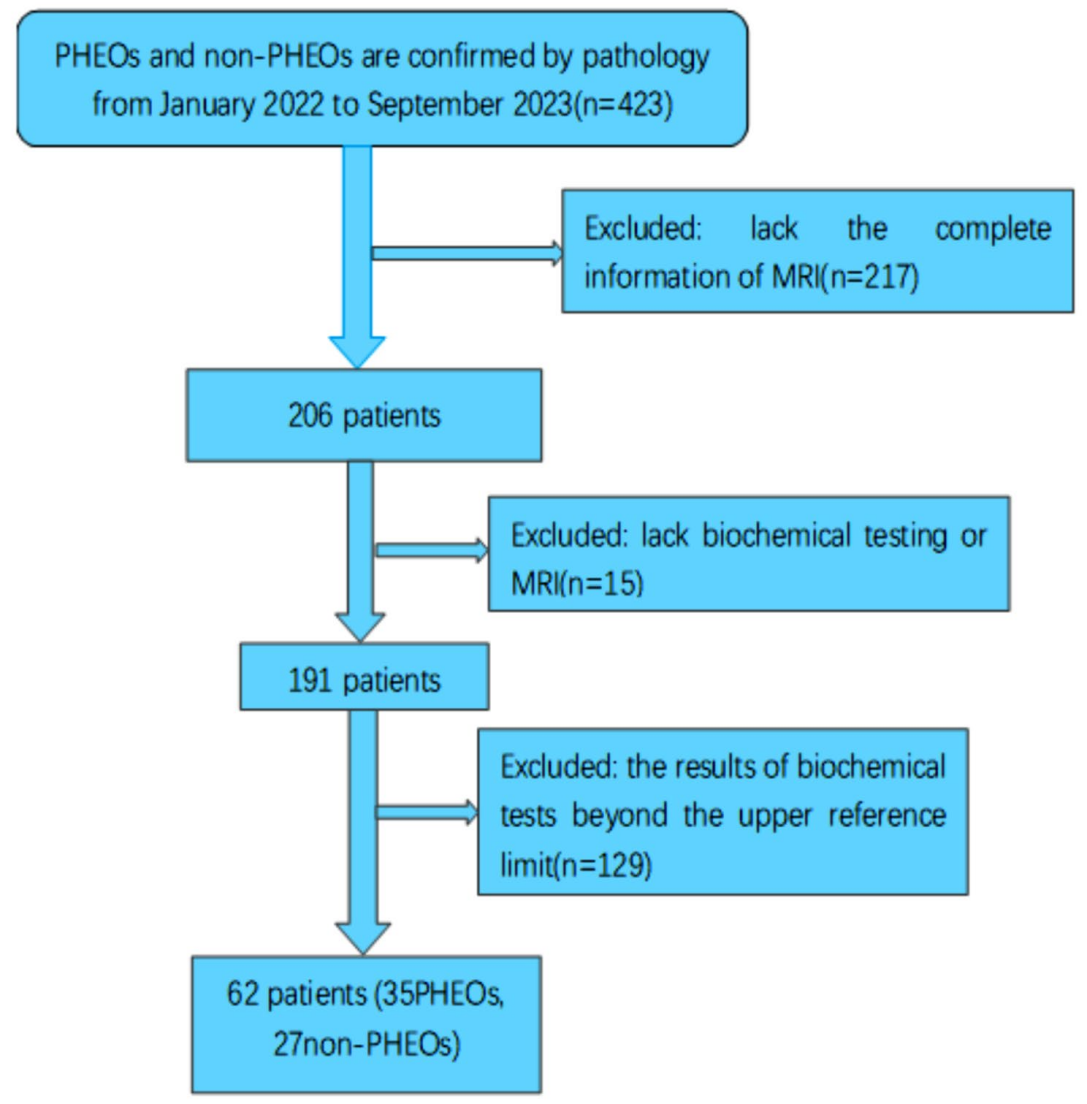
Flowchart shows exclusion criteria for the study. PHEOs, pheochromocytomas. non-PHEOs, non- pheochromocytomas

This retrospective study was approved by the Institutional Ethical Committees of the Affiliated Drum Tower Hospital of Medical School of Nanjing University, and a waiver of informed consent was granted. We finally identified 62 patients (35PHEOs, 27non-PHEOs) who met the inclusion criteria between January 2022 to September 2023. Inclusion criteria were as follows: (1) final diagnosis of adrenal tumors documented by histology(*n* = 423), (2) the complete information of multiparameter magnetic resonance(*n* = 206), (3) received biochemical screening tests of catecholamines and/or catecholamine metabolites (*n* = 191), (4) the results of biochemical screening tests beyond the upper reference limit (*n* = 62). Therefore, this study was focused on MRI imaging values. Figure 1 shows the flow chart of the study profile.

### MRI technique

MRI was performed at a single tertiary care referral center using one of three clinical 3-T systems (Symphony or TRIO, Siemens Healthcare or Discovery 750 W, General Electric Healthcare). All prebiopsy magnetic resonance images, consisting of T1 weighted imaging, T2-weighted imaging and T2-SPAIR (Spectral Attenuated Inversion Recovery) imagine, diffusion-weighted imaging, and Apparent Diffusion Coefficient (ADC), (HASTE, Siemens Healthcare and SSFSE, GE Healthcare).

### MRI image analysis

**Fig. 2:**
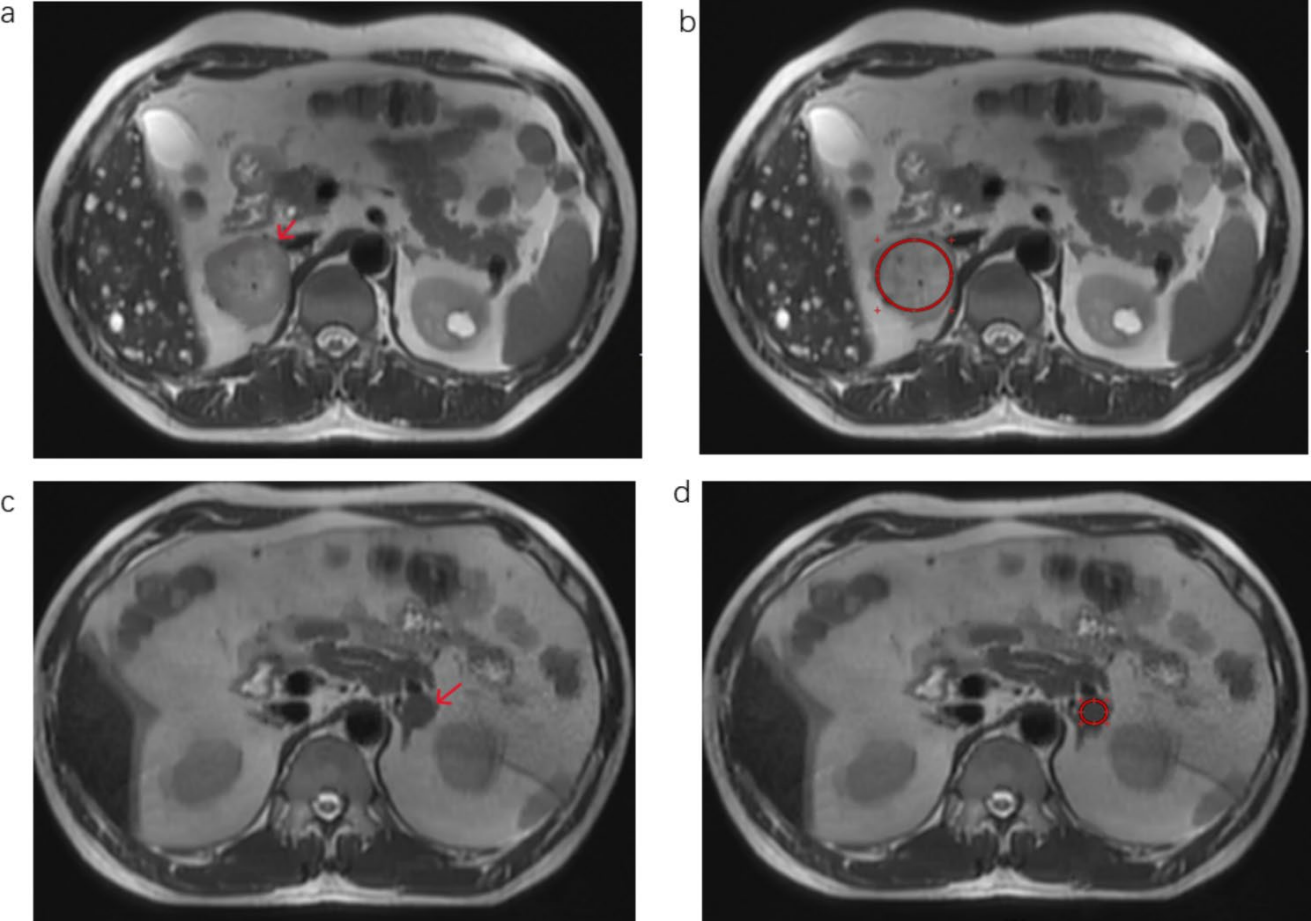
MR images in a man with right 5.2 cm pheochromocytoma(**a-b**) and in a man with left 1.7 cm adrenal adenoma (c-d). (**a**) Axial T2-weighted single-shot turbo spin-echo image depicts the right adrenal nodule (arrow) with high signal intensity (SI). (**c**) Axial T2-weighted single-shot turbo spin-echo image depicts the left adrenal nodule (arrow) with low signal intensity (SI). (**b**, **d**) shows method of measurement of T2-weighted SI ratio. Circular (red) region of interest (ROI) depicts method of measurement of adrenal T2-weighted SI for homogeneous nodules. A ROI was placed in the ipsilateral skeletal muscle (not shown) to measure the adrenal-to-muscle T2-weighted SI ratio

Three radiologists, blinded to the pathologic diagnosis reviewed all existing MRI images in each patient, respectively and independently, the first radiologist with 5 years of experience, and the second radiologist with 14 years of experience, and the last radiologist with 18 years of experience. Disagreements regarding image analyses were worked out by consensus. The following multiparameter MRI findings were recorded: (a) size: long (LD) and short (SD) diameters; (b) axial in-phase [IP] and opposed-phase [OP] T1-weighted images signal intensity (SI); (c) axial T2-weighted images and T2-SPAIR signal intensity (SI); (c) diffusion-weighted imaging (DWI, b1000) signal intensity; (d) the value of Apparent Diffusion Coefficient (ADC). Measurements were performed on axial T2-weighted images, by three radiologists, utilizing the largest central slice of the lesion. For homogeneous lesions, we performed a circular region of interest (ROI) within the nodule to encompass as large as the adrenal nodule for the purpose of covering the entire size of the lesion as far as possible. (Fig. 2). In addition, as for heterogeneous tumors, a circular ROI was also performed within the lesion, involving most of the T2W hyperintensity of the nodule By subjective judgment, and measured Range size of at least 5 mm diameter, as previously reported [28], to explain the potential average of SI values of heterogeneous nodules. A fixed diameter (5 mm) size ROI was located in the skeletal muscle on the same side to measure T2W muscle SI, allowing the ratio of T2W nodule to muscle SI to be counted (SI nodule /SI muscle) [29]. The skeletal muscle could be served as an internal reference criterion on T2W-MRI since calculations could be performed at approximately the same anteroposterior level as the adrenal nodules to minimize variations associated with the design of the receiver coil [30]. Measurements for T2-SPAIR, T1-IP, T1-OP, DWI (b1000), ADC, a circular ROI was placed in the nodule as was performed for T2-weighted images.; As for chemical shift (in-phase [IP] and opposed-phase [OP]). A fixed diameter (5 mm) ROI was also placed in the spleen to measure T1-IP spleen SI and T1-OP spleen SI so that the chemical shift adrenal-to-spleen (ASR) SI ratio could be calculated [31, 32]. ASR = (SI lesion OP/SI spleen OP)/ (SI lesion IP/SI spleen IP).

### Statistical analysis

All statistical data analyses are performed with SPSS (version 25.0, SPSS, Chicago, IL). In order to determine statistical significance, a commonly accepted threshold of *P* < 0.05 was employed. Continuous data were present as mean ± standard deviation (with range provided); however, for categorical variables are expressed as a proportion. Independent t-tests were used for comparison of normally distributed continuous data, while Mann-Whitney U tests were implemented for non-normally distributed continuous data. The chi-square test was applied to compare proportions and diagnostic accuracy using 2 × 2 tables for categorical variables. A receiver operating characteristic (ROC) analysis was used to determine diagnostic accuracy. The cutoff values of the maximum sensitivity and specificity at the maximal value of the Youden’s index were obtained by operating ROC. Significant statistical variables on univariate analysis were included as available factors in the logistic regression analysis to determine the finally significant parameter.

## Result

### Patient overview

Patient demographic characteristics are summarized in Table 1. As can be seen from this table, the size of lesions with pheochromocytoma have significant difference compared to those non-pheochromocytoma (LD: 39.83 ± 19.23 mm versus 24.19 ± 9.43 mm; *p* = 0.001; SD: 32.03 ± 13.71 mm versus 21.19 ± 8.69 mm, *p* = 0.002), according to MRI lesions evaluation. The age of patients with pheochromocytoma (49.8 years ± 14.42) were younger compared to that adrenal adenoma (57.2 years ± 9.80), *p* = 0.024, but the difference was not significant. Otherwise, there is no difference in laterality, BMI, hypertension, diabetes, cardiovascular events and cerebrovascular events between PHEOs and non-PHEOs.

**Table 1:** Baseline characteristics of patients with PHEOs and non-PHEOs

	PHEO(*n* = 35)	non-PHEO(*n* = 27)	*P* value
Age(years)	49.8 ± 14.42	57.2 ± 9.80	0.024*
**Gender**			
Men	20(57.1%)	13(48.1%)	0.482▾
Women	15(42.9%)	14(51.9%)	
Weight(kg)	62.63 ± 12.80	66.50 ± 9.24	0.190*
BMI (kg/m2)	22.56 ± 3.18	24.98 ± 2.80	0.619*
Hypertension	21(60.0%)	22(81.5%)	0.069▾
Diabetes	*7(20%)*	5(18.5%)	0.884▾
Cerebrovascular events	2(5.7%)	3(11.1%)	0.762▾
Cardiovascular events	2(5.7%)	1(3.7%)	1.000▾
LD(mm)	39.83 ± 19.23	24.19 ± 9.43	0.001*
SD(mm)	32.03 ± 13.71	21.19 ± 8.69	0.002*
LD/SD	1.24 ± 0.25	1.25 ± 0.0.15	0.900*
**Laterality**			
Left	16(45.7%)	17(63%)	0.177▾
Right	19(54.3%)	10(37%)	

Numbers of patients in each group are indicated in brackets. *Data are means ± standard deviation, and the statistical values are the independent sample t-test results▾Data in parentheses are percentages, and the statistical values are the chi-square test results. PHEOs, pheochromocytomas. non-PHEOs, non- pheochromocytomas

### Univariate analyses of the imaging characteristics

According to univariate analyses, we can see that T1 in-phase (T1-IP) and opposed-phase (T1-OP) and diffusion-weighted imaging (DWI, b1000) signal intensity have no significant difference between PHEOs and non-PHEOs (*p* = 0.185; *p* = 0.924; *p* = 0.141, respectively). In addition, T2-weighted signal intensity (T2SI) and T2-SPAIR signal intensity, and the value of ADC in PHEOs are higher than non-PHEOs (*p* = 0.001, 95%CI: 1.003–1.010; *p* = 0.003, 95%CI: 1.002–1.010; *p* = 0.001, 95%CI: 1.001–1.005, respectively). Moreover, significant differences are observed in the chemical shift (13.95 ± 11.03 versus 56.07 ± 54.04, *p* < 0.001), chemical shift index (11.54 ± 6.85, versus 35.40 ± 20.19, *p* < 0.001) and T2SI-ratio (3.33 ± 1.38 versus 1.77 ± 0.37, *p* < 0.001). However, there was no difference in adrenal-to-spleen chemical shift SI ratio (ASR) (0.820 ± 1.100, versus 0.771 ± 0.270, *p* = 0.335). Univariate analyses of the imaging paraments are summarized in Table 2.

**Table 2:** Univariable analyses of MRI features between PHEOs and non‑PHEOs

Variables	PHEO (*n* = 35)	non-PHEO(*n* = 27)	*P* value	OR	95%CI
T1-IP	129.63 ± 97.29	169.63 ± 128.39	0.185	0.997	0.992–1.002
T1-OP	115.69 ± 90.65	113.30 ± 110.47	0.924	1.000	0.995–1.005
Chemical shift	13.95 ± 11.03	56.07 ± 54.04	< 0.001	0.932	0.897–0.968
Chemical shift index	11.54 ± 6.85	35.40 ± 20.19	< 0.001	0.884	0.830–0.942
ASR	0.820 ± 1.100	0.771 ± 0.270	0.335	3.682	0.261–52.012
T2SI	657.80 ± 250.40	417.56 ± 149.21	0.001	1.006	1.003–1.010
T2SI-ratio	3.33 ± 1.38	1.77 ± 0.37	< 0.001	20.553	3.881-108.837
T2-spair	478.80 ± 217.61	310.96 ± 147.68	0.003	1.007	1.002–1.010
ADC	1515.51 ± 441.25	1154.93 ± 188.62	0.001	1.003	1.001–1.005
b1000	305.11 ± 165.53	248.48 ± 112.82	0.141	1.003	0.999–1.007

Imaging features of PHEO and non-PHEO lesions. Data are means ± standard deviation. *p* < 0.05 indicate a significant difference between lesions. T1-IP, T1-weighted images in-phase signal intensity; T1-OP, T1-weighted images opposed-phase signal intensity; Chemical shift, T1-IP subtract T1-OP; T2SI: T2-weighted images signal intensity; Chemical shift index, (T1-IP–T1-OP)/ T1-IP*100; ASR = (SI lesion OP/SI spleen OP)/ (SI lesion IP/SI spleen IP); T2-weighted images signal intensity; T2SI-ratio, T2SI nodule /T2SI muscle

### Binary logistic regression analysis

**Fig. 3:**
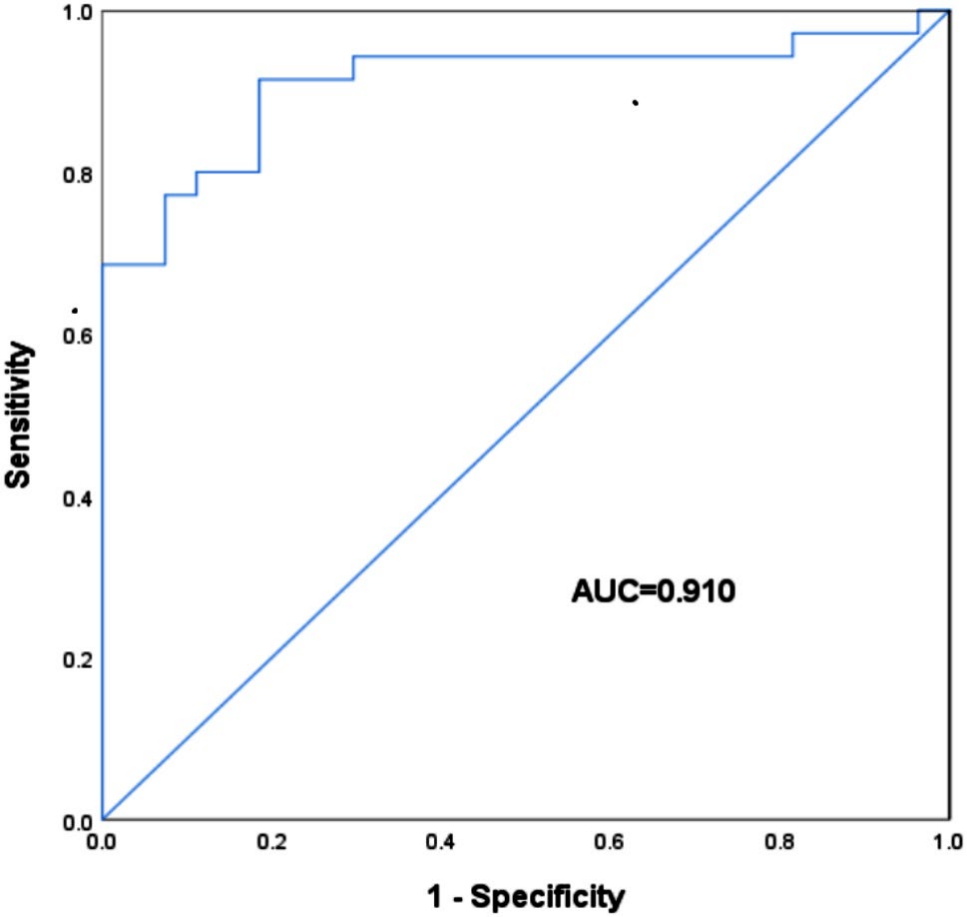
ROC of T2SI-ratio for differentiating PHEOs from nonPHEOs. The AUC was 0.910 (95% CI: 0.833–0.987), with sensitivity, specificity of 91.4%, 81.5%, respectively

Based on the univariate analysis result, six variables (chemical shift, chemical shift index, T2SI, T2SI radio, T2-spair and ADC) were involved in the binary logistic regression analysis (Table 3). Ultimately, the result of statistical significance was only found in T2SI ratio for distinguishing PHEOs from non-PHEOs (*p* = 0.035, 95% CI: 1.151–42.757, OR = 7.016). ROC analysis was performed of the quantitative variables, we established that T2SI ratio ( > = 2.01) was independent predictive factor for differentiating PHEOs. Area under ROC curve (AUC) for diagnosis of PHEOs utilizing T2-weighted SI ratio evaluated independently was 0.910 (95% CI: 0.833–0.987) (Fig. 3). At the maximal value of the Youden’s index (0.729), the maximum sensitivity and specificity were 91.4% and 81.5%, respectively. The sensitivity, specificity, and accuracy for diagnosis of pheochromocytoma are summarized in Table 4.

**Table 3:** Multivariable logistic regression analysis for identifying PHEOs

Variables	B	Wald	*P* value	OR	95%CI
Chemical shift	-0.045	1.161	0.281	0.956	0.881–1.037
Chemical shift index	-0.022	0.117	0.732	0.978	0.862–1.110
T2SI	0.000	0.012	0.912	1.000	0.994–1.006
T2SI-ratio	1.948	4.463	0.035	7.016	1.151–42.757
T2-spair	0.004	1.048	0.306	1.004	0.996–1.012
ADC	-0.001	0.096	0.757	0.999	0.996–1.003

OR = odds ratio; CI = confidence interval

**Table 4:** Diagnostic accuracy of the MRI features for diagnosis of pheochromocytomas

	Sensitivity (95% confidence intervals)	Specificity (95% confidence intervals)	Overall accuracy (95% confidence intervals
Chemical shift	0.889	0.800	0.856
Chemical shift index	0.815	0.886	0.880
T2SI	0.743	0.815	0.803
T2SI-ratio	0.914	0.815	0.910
T2-spair	0.714	0.889	0.800
ADC	0.686	0.889	0.772

## Discussion

Though biochemical testing is recommended for the workup of incidental adrenal nodules with reported accuracy of over 90% for diagnosis of pheochromocytoma [3, 10, 33], these tests may be falsely positive [12]. The specificity of the detection of catecholamines and their metabolites depends largely on preanalytical criteria, which are susceptible to some drugs (e.g., dopamine D2-receptor antagonists), and the need for proper collection of 24-hour urine and blood samples [34]. Therefore, the measurement of epinephrine is quite expensive and demanding. In addition, although PET-CT can be highly sensitive to observe pheochromocytoma and paraganglioma, and in the case of malignant tumors, it can show metastasis [35]. However, due to the high cost of the examination and the difficulty in promoting it in primary hospitals, its application is limited. To differentiate the PHEOs from incidental adrenal nodules, especially the result of biochemical testing is positive, we investigate the magnetic resonance imaging characteristics. In our study, there is distinct significance in tumor size (LD: 39.83 ± 19.23 mm versus 24.19 ± 9.43 mm; *p* = 0.001) among PHEOs from non-PHEOs, although we didn’t include it in the final logistic regression analysis. On one hand, pheochromocytomas demonstrated higher T2W and T2-spair signal intensity compared to adenoma, similar to what has been published previously [36]. Varghese et al. were the first to assess adrenal lesions SI at T2-weighted MRI comparing pheochromocytomas and adenomas, showing that a majority of adenomas showed low T2-weighted signal [27]. As we all know that very high T2W signal intensity is a representative characteristic of pheochromocytoma; nevertheless, approximately 30% of pheochromocytomas are not bright on T2W [27]. Besides, pheochromocytoma may show atypical features when cystic, hemorrhagic, and necrotic lesions occur, making it difficult to distinguish it from other adrenal tumors [37, 38]. Maurea et al. [39]. systematically studies the typical or atypical MRI features of PHEOs, the results of their study found that more than half (54%) of PHEOs patients with atypical lesions, and the atypical lesions were mostly cystic. In addition, most of the typical PHEOs indicated no signal loss in T1 chemical shift, nevertheless may increase heterogeneity because of varying degrees of lesion. Furthermore, radionuclide imaging associated with MRI was also used to compare typical and atypical pheochromocytoma features [40, 41]. The findings showed MRI imaging features similar to the atypical PHEOs described above, and the uptake of non-pheochromocytomas on metaiodobenzylguanidine(MIBG) and fluorine-deoxy-glucose(FDG)-PET was additionally explored. Moreover, the results of research suggested that residual MIBG uptake reminder the characteristics and diagnosis of PHEOs, whereas residual FDG cumulation usually indicates the presence of an active solid composition in neoplastic lesion. On the other hand, T2-weighted SI ratio was also significantly higher in PHEOs adenomas compared with non-PHEOs, independent predictive factor for differentiating PHEOs. Moreover, for chemical shift and chemical shift ratio, significant differences obtained to distinct pheochromocytomas from adrenal nodules in our study. Our study is concordant with what has been proved previously [42]. Our study suggested that the values of ADC might add helpful information in differentiating pheochromocytomas from non-pheochromocytomas. Adenomas indicate with low ADC values on MRI which was attributed to lipid content and intensive packed cells [43, 44]. Furthermore, using different radioactive marker compounds of adrenal nuclide scan can display the corresponding biological pathways, is advantageous to the characterization of adrenal masses, with morphological imaging diagnosis of complementary information [45]. Therefore, the related research conducted by nuclear medicine used in indicating the biological functions of different radionuclides in the role of adrenal characterization and diagnosis, especially in patients with hypersecretory and non-hypersecretory adrenal tumors [46–49]. The results illustrated that nor-cholesterol uptake was highly specific in adenomas, with a positive uptake rate of 100% both in hypersecretory and hypersecretory adenomas; similarly, MIBC uptake was highly specific in pheochromocytoma [47, 49]. In addition, FDG and MIBC also have high capacity to distinguish benign and malignant adrenal tumors, such as carcinoma, sarcoma [48, 49]. However, despite the high specificity of radionuclides, false positives can occur, two and one false positive results were found for non-cholesterol and MIBC, respectively, in the previous investigation [46]. Furthermore, it may be limited to use by the absence of available radioactive material and nuclear facility.

Our study specifically evaluated a common clinical problem, which is the management of an incidentally discovered adrenal nodule in a patient that shows the result of biochemical testing beyond the upper reference limit. The most efficient variable was selected to differentiate PHEOs from adrenal incidentalomas by logistic regression analysis, with the high sensitivity, specificity and accuracy. The results of our study have a high accuracy, which can be used to fill the shortage of catecholamine detection accuracy that is easily affected by external influences. Alternatively, it may be combined with biochemical tests to improve the diagnostic accuracy of pheochromocytoma in clinical practice. In addition, MRI can be performed in a clinical setting that reduces radiation exposure during regular repeat imaging in patients with tumor-predisposition gene mutations (e.g., children, young women of childbearing age). Therefore, it can reduce some unnecessary invasive or expensive and cumbersome examinations in the diagnosis of pheochromocytoma in clinical practice. However, our present study has some limitations that need to be addressed in future studies. First, the number of samples included is limited, which might limit the capacity of the data analyses. It may be that inclusion criteria are quite strict, received MRI and biochemical testing must be present is positive。However, only patients with pathological diagnosis have been included in the investigation, which is also considered a strength. Second, it was a single-center retrospective study that may have been subject to selection bias, may limit broader applicability of our results; verification of our results in other institutions is necessary. Third, we didn’t compare the effects of heterogeneity on them, although attempted to reduce impact by measuring methods. Moreover, three radiologists are blinded to the pathologic diagnosis reviewed all existing MRI images to reduce differences. However, we included more magnetic resonance parameters to study the characteristics of magnetic resonance imaging to differentiate pheochromocytomas among adrenal tumors more comprehensively compared to previous studies. Future research should include a great deal of samples to evaluate for potential improvements in accuracy by adding additional variables (including size and demographic variables) into logistic regression analysis.

In conclusion, our study demonstrates that the T2-weighted SI ratio, with highly sensitivity, specificity, and overall accuracy to differentiate adrenal pheochromocytomas from adrenal tumors. Pheochromocytoma is badly dangerous and needs to be treated with caution. Therefore, it is crucial to identify PHEOs accurately, when the outputs of biochemical testing exceed the upper limit of the reference value. The results of this study can be used as a complementary method to improve the diagnostic accuracy of pheochromocytoma. These observations and propose thresholds are required to validate in the future multicenter studies, which could enable diagnosis in clinical practice.

### Electronic supplementary material

Below is the link to the electronic supplementary material.

Supplementary Material 1

Supplementary Material 2

Supplementary Material 3

## Data Availability

All data supporting the conclusions of this article will be made available by the authors; further enquiries can be directed to the corresponding authors.

## References

[1] Young WFJ. Clinical practice.The incidentally discovered adrenal mass. N Engl J Med. 2007;356(6):601–10. 17287480 10.1056/NEJMcp065470

[2] Glazer DI, Mayo-Smith WW. Management of incidental adrenal masses: an update. Abdom Radiol. 2020;45(4):892–900. 10.1007/s00261-019-02149-2 31359097

[3] Fassnacht M, Arlt W, Bancos I, Dralle H, Newell-Price J, Sahdev A, Tabarin A, Terzolo M, Tsagarakis S, Dekkers OM. Management of adrenal incidentalomas: European Society of Endocrinology Clinical Practice Guideline in collaboration with the European Network for the study of adrenal tumors. Eur J Endocrinol. 2016;175(2):G1–34. 27390021 10.1530/EJE-16-0467

[4] Araujo-Castro M, Iturregui Guevara M, Calatayud Gutierrez M, Parra Ramirez P, Gracia Gimeno P, Hanzu FA, Lamas Oliveira C. Practical guide on the initial evaluation, follow-up, and treatment of adrenal incidentalomas adrenal diseases Group of the Spanish Society of Endocrinology and Nutrition. Endocrinol Diabetes Nutr (Engl Ed). 2020;67(6):408–19. 32349941 10.1016/j.endinu.2020.03.002

[5] Young WF. Management approaches to adrenal incidentalomas - a view from Rochester, Minnesota. Endocrin Metab Clin. 2000;29(1):159–. 10.1016/S0889-8529(05)70122-5 10732270

[6] Motta-Ramirez GA, Remer EM, Herts BR, Gill IS, Hamrahian AH. Comparison of CT findings in symptomatic and incidentally discovered pheochromocytomas. Am J Roentgenol. 2005;185(3):684–8. 16120918 10.2214/ajr.185.3.01850684

[7] Paschou SA, Vryonidou A, Goulis DG. Adrenal incidentalomas: a guide to assessment, treatment and follow-up. Maturitas. 2016;92:79–85. 27621243 10.1016/j.maturitas.2016.07.017

[8] Stolk RF, Bakx C, Mulder J, Timmers HJLM, Lenders JWM. Is the excess Cardiovascular morbidity in Pheochromocytoma related to blood pressure or to catecholamines? J Clin Endocr Metab. 2013;98(3):1100–6. 23408574 10.1210/jc.2012-3669

[9] Khorram-Manesh A, Ahlman H, Nilsson O, Odén A, Jansson S. Mortality associated with pheochromocytoma in a large Swedish cohort. Ejso. 2004;30(5):556–9. 15135486 10.1016/j.ejso.2004.03.006

[10] Kebebew E. Adrenal incidentaloma. N Engl J Med. 2021;384(16):1542–51. 33882207 10.1056/NEJMcp2031112

[11] Bokuda K, Yatabe M, Seki Y, Ichihara A. Clinical factors affecting spot urine fractionated metanephrines in patients suspected pheochromocytoma/paraganglioma. Hypertens Res. 2020;43(6):543–9. 32020083 10.1038/s41440-020-0406-4

[12] Sawka AM, Prebtani AP, Thabane L, Gafni A, Levine M, Young WF Jr. A systematic review of the literature examining the diagnostic efficacy of measurement of fractionated plasma free metanephrines in the biochemical diagnosis of pheochromocytoma. BMC Endocr Disord. 2004;4(1):2. 15225350 10.1186/1472-6823-4-2 PMC459231

[13] Kang S, Oh YL, Park SY. Distinguishing pheochromocytoma from adrenal adenoma by using modified computed tomography criteria. Abdom Radiol. 2021;46(3):1082–90. 10.1007/s00261-020-02764-4 32951125

[14] Mohammed MF, ElBanna KY, Ferguson D, Harris A, Khosa F. Pheochromocytomas Versus Adenoma: role of venous phase CT enhancement. Am J Roentgenol. 2018;210(5):1073–8. 29570377 10.2214/AJR.17.18472

[15] Northcutt BG, Raman SP, Long C, Oshmyansky AR, Siegelman SS, Fishman EK, Johnson PT. MDCT of adrenal masses: can dual-phase enhancement patterns be used to Differentiate Adenoma and Pheochromocytoma? Am J Roentgenol. 2013;201(4):834–9. 24059372 10.2214/AJR.12.9753

[16] Northcutt BG, Trakhtenbroit MA, Gomez EN, Fishman EK, Johnson PT. Adrenal adenoma and pheochromocytoma: comparison of multidetector CT venous enhancement levels and washout characteristics. J Comput Assist Tomo. 2016;40(2):194–200. 10.1097/RCT.0000000000000343 26978001

[17] Patel J, Davenport MS, Cohan RH, Caoili EM. Can established CT attenuation and washout criteria for adrenal adenoma accurately exclude pheochromocytoma? Am J Roentgenol. 2013;201(1):122–7. 23789665 10.2214/AJR.12.9620

[18] Woo S, Suh CH, Kim SY, Cho JY, Kim SH. Pheochromocytoma as a frequent false-positive in adrenal washout CT: a systematic review and meta-analysis. Eur Radiol. 2018;28(3):1027–36. 29026974 10.1007/s00330-017-5076-5

[19] Park BK, Kim CK, Kim B, Lee JH. Comparison of delayed enhanced CT and chemical shift MR for evaluating hyperattenuating incidental adrenal masses. Radiology. 2007;243(3):760–5. 17517932 10.1148/radiol.2433051978

[20] Seo JM, Park BK, Park SY, Kim CK. Characterization of lipid-poor adrenal adenoma: Chemical-Shift MRI and Washout CT. Am J Roentgenol. 2014;202(5):1043–50. 24758658 10.2214/AJR.13.11389

[21] Park BK, Kim CK, Kwon GY, Kim JH. Re-evaluation of pheochromocytomas on delayed contrast-enhanced CT: washout enhancement and other imaging features. Eur Radiol. 2007;17(11):2804–9. 17549484 10.1007/s00330-007-0695-x

[22] Park BK, Kim B, Ko K, Jeong SY, Kwon GY. Adrenal masses falsely diagnosed as adenomas on unenhanced and delayed contrast-enhanced computed tomography: pathological correlation. Eur Radiol. 2006;16(3):642–7. 16215735 10.1007/s00330-005-0017-0

[23] Bilbey JH, Mcloughlin RF, Kurkjian PS, Wilkins GEL, Chan NHL, Schmidt N, Singer J. Mr-Imaging of adrenal masses - Value of Chemical-Shift Imaging for distinguishing adenomas from other tumors. Am J Roentgenol. 1995;164(3):637–42. 7863885 10.2214/ajr.164.3.7863885

[24] Mitchell DG, Crovello M, Matteucci T, Petersen RO, Miettinen MM. Benign Adrenocortical masses - diagnosis with Chemical-Shift Mr Imaging. Radiology. 1992;185(2):345–51. 1410337 10.1148/radiology.185.2.1410337

[25] Schieda N, Siegelman ES. Update on CT and MRI of adrenal nodules. Am J Roentgenol. 2017;208(6):1206–17. 28225653 10.2214/AJR.16.17758

[26] Schieda N, Alrashed A, Flood TA, Samji K, Shabana W, McInnes MDF. Comparison of quantitative MRI and CT Washout Analysis for differentiation of adrenal pheochromocytoma from adrenal adenoma. Am J Roentgenol. 2016;206(6):1141–8. 27011100 10.2214/AJR.15.15318

[27] Varghese JC, Hahn PF, Papanicolaou N, MayoSmith WW, Gaa JA, Lee MJ. MR differentiation of phaeochromocytoma from other adrenal lesions based on qualitative analysis of T2 relaxation times. Clin Radiol. 1997;52(8):603–6. 9285420 10.1016/S0009-9260(97)80252-8

[28] Tu W, Abreu-Gomez J, Udare A, Alrashed A, Schieda N. Utility of T2-weighted MRI to differentiate adrenal metastases from lipid-poor adrenal adenomas. Radiol-Imag Cancer 2020, 2(6). 10.1148/rycan.2020200011 PMC7983803 33778748

[29] Small (<4 cm) Renal Mass: Differentiation of Angiomyolipoma without Visible Fat from Renal Cell Carcinoma Utilizing MR Imaging. *Radiology* 2012, 263(1):160–168. 10.1148/radiol.12111205 22344404

[30] Schieda N, Coffey N, Gulavita P, Al-Dandan O, Shabana W, Flood TA. Prostatic ductal adenocarcinoma: an aggressive tumour variant unrecognized on T2 weighted magnetic resonance imaging (MRI). Eur Radiol. 2014;24(6):1349–56. 24687527 10.1007/s00330-014-3150-9

[31] Karlo CA, Donati OF, Burger IA, Zheng JT, Moskowitz CS, Hricak H, Akin O. MR imaging of renal cortical tumours: qualitative and quantitative chemical shift imaging parameters. Eur Radiol. 2013;23(6):1738–44. 23300041 10.1007/s00330-012-2758-x

[32] Hindman N, Ngo L, Genega EM, Melamed J, Wei J, Braza JM, Rofsky NM, Pedrosa I. Angiomyolipoma with Minimal Fat: can it be differentiated from Clear Cell Renal Cell Carcinoma by using standard MR techniques? Radiology. 2012;265(2):468–77. 23012463 10.1148/radiol.12112087 PMC3480813

[33] Young WF Jr. Clinical practice. The incidentally discovered adrenal mass. N Engl J Med. 2007;356(6):601–10. 17287480 10.1056/NEJMcp065470

[34] Därr R, Kuhn M, Bode C, Bornstein SR, Pacak K, Lenders JWM, Eisenhofer G. Accuracy of recommended sampling and assay methods for the determination of plasma-free and urinary fractionated metanephrines in the diagnosis of pheochromocytoma and paraganglioma: a systematic review. Endocrine. 2017;56(3):495–503. 28405881 10.1007/s12020-017-1300-y PMC6297899

[35] Sadowski SM, Millo C, Cottle-Delisle C, Merkel R, Yang LA, Herscovitch P, Pacak K, Simonds WF, Marx SJ, Kebebew E. Results of (68)Gallium-DOTATATE PET/CT scanning in patients with multiple endocrine neoplasia type 1. J Am Coll Surg. 2015;221(2):509–17. 26206648 10.1016/j.jamcollsurg.2015.04.005 PMC4515773

[36] Gerson R, Tu W, Abreu-Gomez J, Udare A, McPhedran R, Ramsay T, Schieda N. Evaluation of the T2-weighted (T2W) adrenal MRI calculator to differentiate adrenal pheochromocytoma from lipid-poor adrenal adenoma. Eur Radiol. 2022;32(12):8247–55. 35680653 10.1007/s00330-022-08867-4

[37] Jacques AET, Sahdev A, Sandrasagara M, Goldstein R, Berney D, Rockall AG, Chew S, Reznek RH. Adrenal phaeochromocytoma: correlation of MRI appearances with histology and function. Eur Radiol. 2008;18(12):2885–92. 18641999 10.1007/s00330-008-1073-z

[38] Leung K, Stamm M, Raja A, Low G. Pheochromocytoma: the Range of appearances on Ultrasound, CT, MRI, and functional imaging. Am J Roentgenol. 2013;200(2):370–8. 23345359 10.2214/AJR.12.9126

[39] Maurea S, Attanasio L, Galatola R, Romeo V, Stanzione A, Camera L, Klain M, Simeoli C, Modica R, Mascolo M, et al. MR imaging characterization of pheochromocytoma: a comparison between typical and atypical tumor lesions. Clin Transl Imaging. 2024;12(3):337–46. 10.1007/s40336-023-00608-x

[40] Galatola R, Romeo V, Simeoli C, Guadagno E, De Rosa I, Basso L, Mainolfi C, Klain M, Nicolai E, Colao A, et al. Characterization with hybrid imaging of cystic pheochromocytomas: correlation with pathology. Quant Imag Med Surg. 2021;11(2):862–9. 10.21037/qims-20-490 PMC7779916 33532285

[41] Galatola R, Attanasio L, Romeo V, Mainolfi C, Klain M, Simeoli C, Modica R, Guadagno E, Aprea G, Basso L et al. Characterization of Atypical Pheochromocytomas with Correlative MRI and Planar/Hybrid Radionuclide Imaging: A Preliminary Study. *Appl Sci-Basel* 2021, 11(20).

[42] Israel GM, Korobkin M, Wang C, Hecht EN, Krinsky GA. Comparison of unenhanced CT and chemical shift MRI in evaluating lipid-rich adrenal adenomas. Am J Roentgenol. 2004;183(1):215–9. 15208141 10.2214/ajr.183.1.1830215

[43] Halefoglu AM, Altun I, Disli C, Ulusay SM, Ozel BD, Basak M. A prospective study on the utility of diffusion-weighted and Quantitative Chemical-Shift Magnetic Resonance Imaging in the distinction of adrenal adenomas and metastases. J Comput Assist Tomo. 2012;36(4):367–74. 10.1097/RCT.0b013e3182597613 22805662

[44] Tsushima Y, Takahashi-Taketomi A, Endo K. Diagnostic utility of Diffusion-Weighted MR Imaging and Apparent Diffusion Coefficient Value for the diagnosis of adrenal tumors. J Magn Reson Imaging. 2009;29(1):112–7. 19097108 10.1002/jmri.21616

[45] Ilias L, Sahdev A, Reznek RH, Grossman AB, Pacak K. The optimal imaging of adrenal tumours: a comparison of different methods. Endocr-Relat Cancer. 2007;14(3):587–99. 17914090 10.1677/ERC-07-0045

[46] Maurea S, Klain M, Caracò C, Ziviello M, Salvatore M. Diagnostic accuracy of radionuclide imaging using I nor-cholesterol or -iodobenzylguanidine in patients with hypersecreting or non-hypersecreting adrenal tumours. Nucl Med Commun. 2002;23(10):951–60. 12352593 10.1097/00006231-200210000-00004

[47] Maurea S, Mainenti PP, Romeo V, Mollica C, Salvatore M. Nuclear imaging to characterize adrenal tumors: comparison with MRI. World J Radiol. 2014;6(7):493–501. 25071890 10.4329/wjr.v6.i7.493 PMC4109101

[48] Maurea S, Cuocolo A, Imbriaco M, Pellegrino T, Fusari M, Cuocolo R, Liuzzi R, Salvatore M. Imaging characterization of benign and malignant pheochromocytoma or paraganglioma: comparison between MIBG uptake and MR signal intensity ratio. Ann Nucl Med. 2012;26(8):670–5. 22752959 10.1007/s12149-012-0624-1

[49] Maurea S, Caraco C, Klain M, Mainolf C, Salvatore M. Imaging characterization of non-hypersecreting adrenal masses - comparison between MR and radionuclide techniques. Q J Nucl Med Mol Im. 2004;48(3):188–97. 15499292

