# Supplementary figures and images for: Diagnostic performance of magnetic resonance imaging features to differentiate adrenal pheochromocytoma from adrenal tumors with positive biochemical testing results
Source: BMC Med Imaging. 2024 Jul 18;24:175. doi: 10.1186/s12880-024-01350-0 (PMC11264621; doi:10.1186/s12880-024-01350-0)

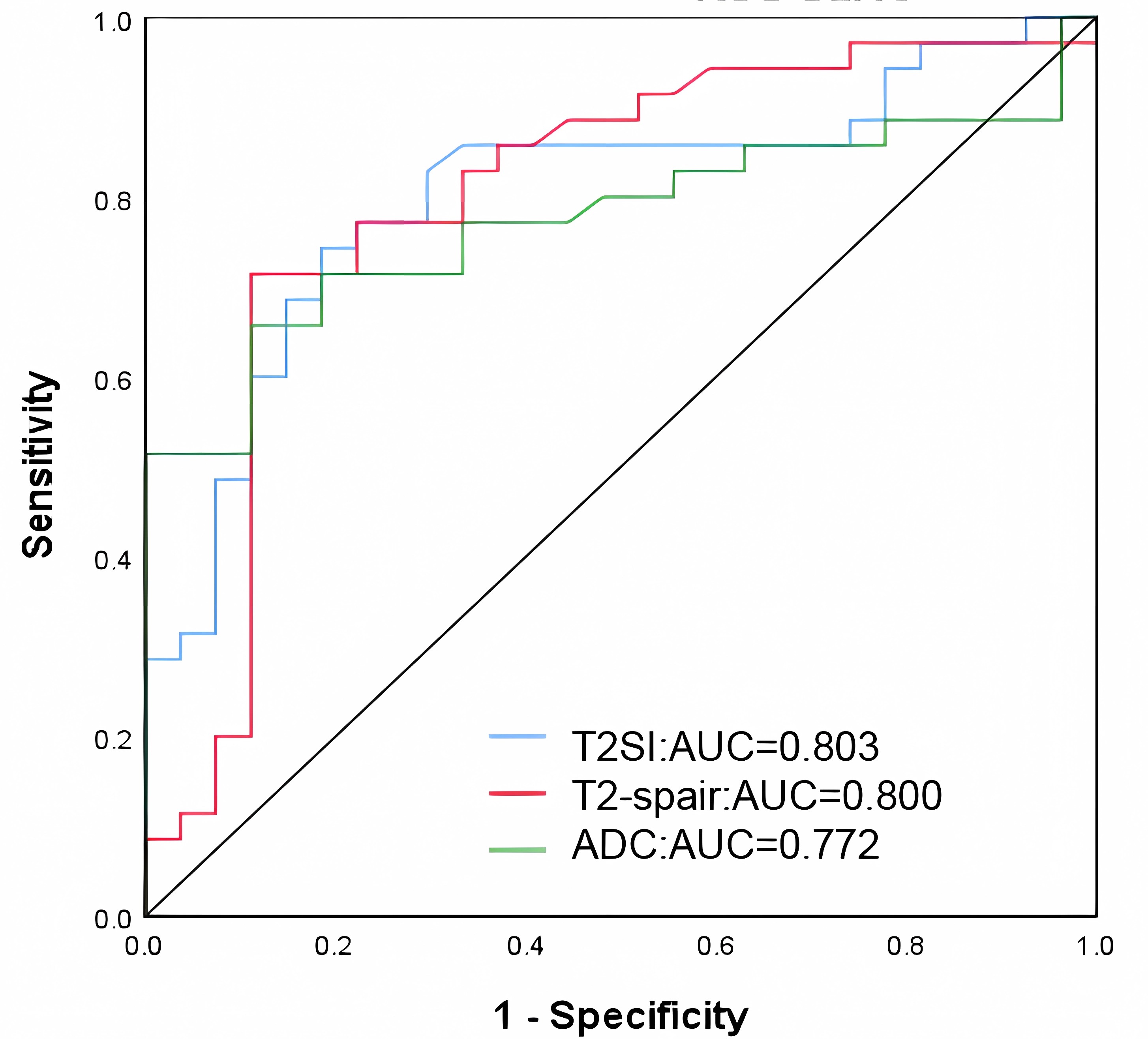

Supplement: Supplementary file 1 — Supplementary Material 1 [file 12880_2024_1350_MOESM1_ESM.jpeg]

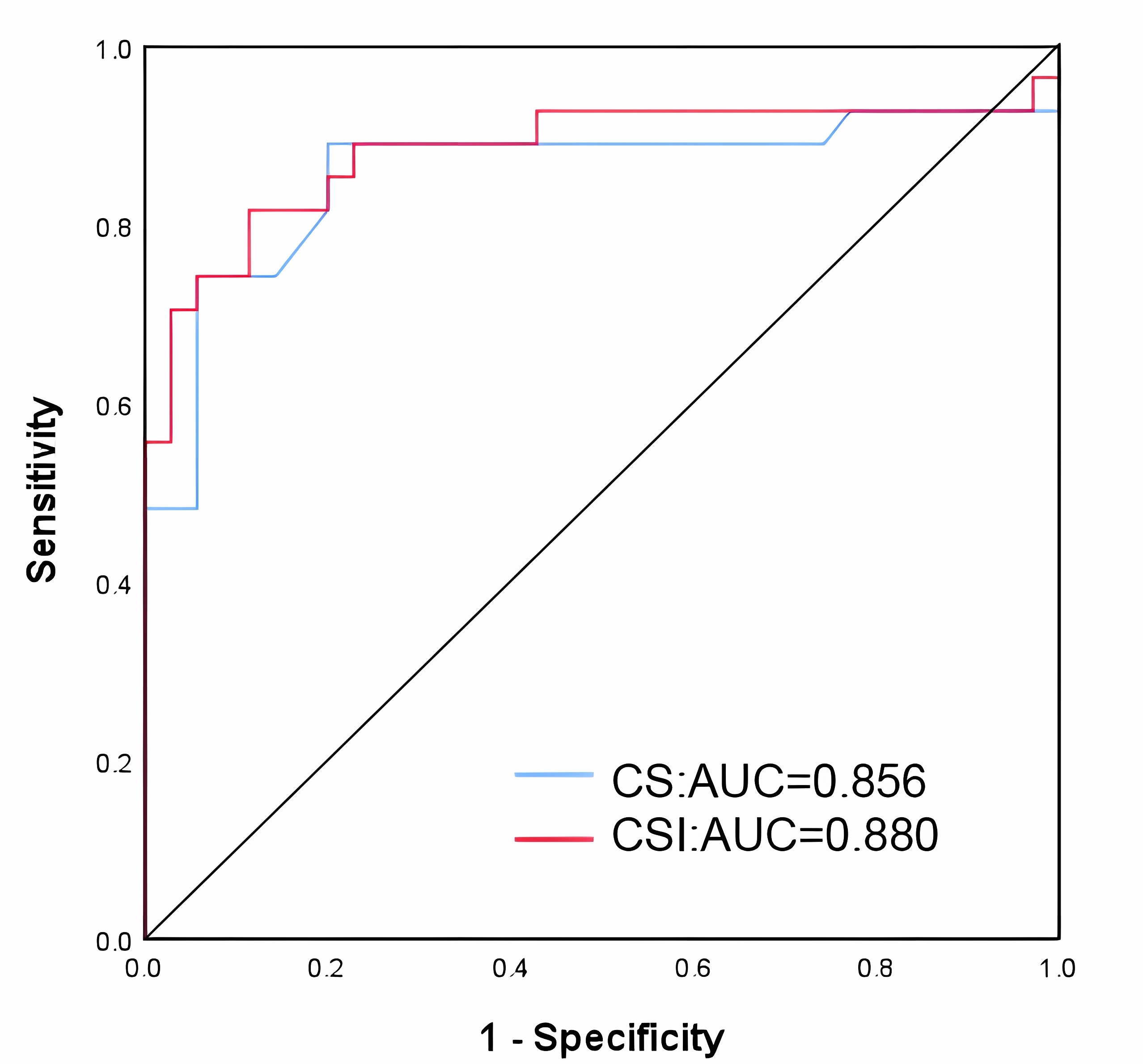

Supplement: Supplementary file 2 — Supplementary Material 2 [file 12880_2024_1350_MOESM2_ESM.jpeg]
