# Supplementary material for: Diagnostic performance of magnetic resonance imaging features to differentiate adrenal pheochromocytoma from adrenal tumors with positive biochemical testing results
Source: BMC Med Imaging. 2024 Jul 18;24:175. doi: 10.1186/s12880-024-01350-0 (PMC11264621; doi:10.1186/s12880-024-01350-0)
Supplement: Supplementary file 3 — Supplementary Material 3 [file 12880_2024_1350_MOESM3_ESM.docx]

**legend of Supplementary Figures**

Supplementary Figure1: ROC of T2SI, T2-spair, and ADC for differentiating PHEOs from non-PHEOs.

Supplementary Figure2: ROC of CS and CSI for differentiating PHEOs from non-PHEOs. CS: Chemical shift; CSI: Chemical shift index.
